# Comparison of Target Features for Predicting Drug-Target Interactions by Deep Neural Network Based on Large-Scale Drug-Induced Transcriptome Data

**DOI:** 10.3390/pharmaceutics11080377

**Published:** 2019-08-02

**Authors:** Hanbi Lee, Wankyu Kim

**Affiliations:** Department of Life Sciences, College of Natural Science, Ewha Womans University, Seoul 03760, Korea

**Keywords:** drug target interaction, deep neural network, drug-induced transcriptome data, drug repositioning

## Abstract

Uncovering drug-target interactions (DTIs) is pivotal to understand drug mode-of-action (MoA), avoid adverse drug reaction (ADR), and seek opportunities for drug repositioning (DR). For decades, in silico predictions for DTIs have largely depended on structural information of both targets and compounds, e.g., docking or ligand-based virtual screening. Recently, the application of deep neural network (DNN) is opening a new path to uncover novel DTIs for thousands of targets. One important question is which features for targets are most relevant to DTI prediction. As an early attempt to answer this question, we objectively compared three canonical target features extracted from: (i) the expression profiles by gene knockdown (GEPs); (ii) the protein–protein interaction network (PPI network); and (iii) the pathway membership (PM) of a target gene. For drug features, the large-scale drug-induced transcriptome dataset, or the Library of Integrated Network-based Cellular Signatures (LINCS) L1000 dataset was used. All these features are closely related to protein function or drug MoA, of which utility is only sparsely investigated. In particular, few studies have compared the three types of target features in DNN-based DTI prediction under the same evaluation scheme. Among the three target features, the PM and the PPI network show similar performances superior to GEPs. DNN models based on both features consistently outperformed other machine learning methods such as naïve Bayes, random forest, or logistic regression.

## 1. Introduction

In silico prediction of drug–target interaction (DTI) is becoming more data-driven than conventional modeling-based approaches such as docking or molecular dynamic simulation. Deep neural network (DNN) is increasingly being applied to highly complex and challenging problems such as protein folding [1]. The vast majority of drugs and compounds are expected to interact with multiple targets, i.e., polypharmacology [2]. While millions of DTIs have been identified, and increasingly keep being revealed, it is still costly and time-consuming to validate DTIs experimentally even by high throughput screening (HTS) [3]. It is most likely that there still exist unknown DTIs for both approved drugs and clinical candidate compounds. Such hidden DTIs could critically impact the drug development process including unexpected clinical outcome, or may broaden their indications through drug repositioning. 

In order to facilitate identification of DTIs, a number of in silico approaches have been developed [3,4,5]. There are three major approaches for compound virtual screening: ligand-based virtual screening (LBVS), structure-based virtual screening (SBVS) such as docking, and the chemogenetic method. Ligand-based approaches are based on the idea that structurally similar compounds tend to have similar binding properties to protein targets. This method requires a sufficient number of known ligand information [6]. SBVS such as docking predicts DTIs by executing the simulation of compound–target interactions based on their 3D structures. Therefore, docking is not or less applicable to protein targets of which 3D structural information is not available or less reliable e.g., homology models based on the poor template proteins of low sequence similarity [7,8]. Chemogenetic approaches integrate heterogeneous information on both compounds and targets, and apply statistical and machine learning techniques including deep neural network (DNN) to predict DTIs [9,10].

The release of Connectivity Map or CMAP, the first large-scale drug-induced transcriptome dataset [11], has led to the identification of a number of drug repositioning candidates for various diseases [12]. CMAP is also shown to be highly useful to elucidate a drug’s modes-of-action (MoA) [13], and infer its unknown targets [14,15,16]. The initial version of CMAP is a collection of ~7000 drug-induced expression profiles (DEPs) in human cancer cell lines treated with 1309 compounds. Recently, CMAP has been extended to the L1000 dataset of Library of Integrated Network-based Cellular Signatures (LINCS) [17], a resource containing 1.3 million gene expression profiles associated with 20,413 chemical perturbagens and ~5000 genetic perturbagens (i.e., single gene knockdown or overexpression). 

Although a number of repositioning drug candidates were discovered by CMAP approach based on inverse pattern matching between drug- and disease-expression signatures [18,19,20,21], it remains a challenge to identify their underlying physical targets. In LINCS dataset, the expression 955 landmark genes (L1000 genes) were actually measured, and the inferred expression levels are provided for additional ~12,000 genes. The expression information of the remaining ~8000 genes are essentially missing, but LINCS dataset still demonstrated its utility in predicting DTIs. Several groups predicted DTIs based on the assumption that drugs of similar DEPs would have common targets [14,15,22]. Schroeder and colleagues predicted targets using the protein–protein interaction (PPI) network based on the observation that drug targets tend to enriched in neighborhood of expression signature genes [16]. Zhang and colleagues constructed DNNs based on both genetically perturbed expression profiles (GEPs) and DEPs [23]. 

In spite of many promising results, these studies were developed and evaluated using different features, and datasets for both training and test, which makes it difficult get an idea of best strategies for further improvement based on comparative analyses. In this work, we investigated how different types of target features affect the performance of DTI prediction based on the same evaluation scheme. We took three representative target features of independent nature, i.e., knockdown GEPs (GEP), PPI network (PPI), and pathway membership (PM), and performed objective comparisons in DTI prediction under a series of parameter combinations. Notably, we tried just conventional design of DNN architectures without any elaborate optimizations because, here, we mainly aim to report the first objective comparison of target features for DTI prediction, which is expected to facilitate development of more innovative approaches. 

## 2. Materials and Methods 

### 2.1. Extraction of Drug Features

We obtained the LINCS L1000 dataset, which includes ~205,034 genes expression profiles perturbed by more than 20 K compounds in 71 human cell lines. LINCS L1000 was generated using Luminex L1000 technology, where the expression levels of 978 landmark genes were measured by fluorescence intensity [17]. The LINCS L1000 dataset provides five different levels of dataset depending on the stage of data processing pipeline. The Level 1 dataset contains the raw expression values from Luminex 1000 platform; the Level 2 dataset gives the gene expression values for 978 landmark genes after deconvolution; the Level 3 provides normalized gene expression values for the landmark genes as well as imputed values for an additional ~12,000 genes; the Level 4 dataset contains z-scores relative to the all the samples or vehicle controls in the plate. The Level 5 dataset is the expression signature genes extracted by merging the z-scores of replicates; We used the Level 5 data marked as *exemplar signatures*, which is relatively more robust, thus reliable set of DEGs (Differentially Expressed Genes). We took the concatenated expression values of 978 landmark genes for both drug-induced expression profiles (DEPs) and their untreated controls, resulting in a vector of 978 + 978 = 1956 in length. Since there are multiple untreated controls, we took the median values of replicates.

### 2.2. Extraction of Target Features

#### 2.2.1. Expression Profiles by Gene Knockdown (GEPs)-Based Target Features

Knockdown GEPs were obtained from LINCS Data Portal (http://lincsportal.ccs.miami.edu/dcic-portal/). The target features based on knockdown GEPs were extracted similarly to the compound features as the concatenated vector of 1956 elements from the landmark GEPs of gene knockdown as well as their control GEPs in LINCS. In order to filter out potentially spurious signals from off-targets, we took only the consensus gene signatures (CGS) for this study. 

#### 2.2.2. Target Features by Protein–Protein Interaction (PPI) Network

Target features were extracted using Node2vec method [24], where vector representation of each node is generated in a given network. The degree of network neighborhood is measured by random walk directed by two parameters, *p* and *q*. The return parameter, *p* and the inout parameter, *q* control the probability of a walk staying inward revisiting nodes or staying close to the preceding nodes (1/*p*), or moving outward farther away (1/*q*). We set the return hyperparameter, *p* = 1, and the inout hyperparameter, *q* = 2. The algorithmic detail of node2vec is available in [24] as well as the source code.

#### 2.2.3. Pathway Membership (PM)-Based Target Features

We extracted vector representations of target genes using Global Vectors for Word Representation (GloVe) method by Pennington et al. [25]. The C2 (curated gene sets) and C5 (Gene Ontology gene sets) from MSigDB (v6.2) were used to define pathway membership [26,27]. GloVe counts global co-occurrence across diverse pathways at different levels, while node2vec extracts local context of network neighborhoods based on random walks. The package for GloVe was downloaded from https://nlp.stanford.edu/projects/glove/.

### 2.3. Construction of Deep Neural Networks (DNNs) and Machine Learning Models

The DNNs were implemented using TensorFlow 1.13 [28] and Keras 2.24 [29]. We adopted Adam [30] to optimize DNNs, and applied dropout = 0.5 on each FC layer [31] & early stopping at 20 epochs in order to prevent overfitting. 

Naïve Bayes (NB), logistic regression (LR), and random forest (RF) methods were implemented using python package, scikit-learn (https://scikit-learn.org). Naïve Bayes was performed under default setting. The LR model was built after experimenting both L1 and L2 regularization methods as well as several values of regularization strength (*C* = 0.01, 0.1 and 1.0). For pathway membership, the LR model was built using L2 regularization at *C* = 0.1 showing best performance among the conditions tested. Similarly, the LR model for PPI was constructed using L2 regularization at *C* = 0.01. For the RF model, the number of max features (*N*) were tested at 1/2 × *F*, *F*, and 2*F*, where *F* = $\sqrt{\mathrm{length}\;\mathrm{of}\;\mathrm{input}\;\mathrm{feature}\;\mathrm{vectors}}$ (default by scikit-learn), where *F* = 47 (=$\sqrt{2\times 978+256}$ = $\sqrt{2\times \mathrm{number}\;\mathrm{of}\;\mathrm{landmark}\;\mathrm{genes}\;\mathrm{for}\;\mathrm{drug}\;\&\;\mathrm{control}+\mathrm{length}\;\mathrm{of}\;\mathrm{target}\;\mathrm{features}\;\mathrm{from}\;\mathrm{PM}\;\mathrm{or}\;\mathrm{PPI}}$). We set *N* = 23 of 2/*F* showing better performance, and generated 10,000 trees in our RF models.

## 3. Results

### 3.1. Extraction of Drug and Target Features

In this work, input features consist of: (i) drug features; and (ii) target features, from which DTI predictions are made for the corresponding drugs and targets. For drug features, we chose to use DEPs of 978 landmark genes from LINCS L1000 because DEP is expected to provide highly rich information on MoA, and have been effectively used for DTI prediction [14,15,16]. DEPs were set common to all the DNN models or other machine learning techniques, so that different target features are compared under the same condition. We used concatenated expression profiles of 978 landmark genes for both drug-treated and untreated controls, which resulted in 978 × 2 = 1956 feature vectors. 

We took three canonical types of target features that represent distinct information on target properties [23,32,33], but were not objectively compared for DTI prediction: (i) knockdown GEPs (GEP); (ii) PPI network (PPI); and (iii) pathway membership (PM). First, knockdown GEPs were available for ~4500 genes across 17 cell lines. Again, GEPs were generated as 1,956 feature vectors by concatenating the two landmark expression profiles by gene knockdown and control. Knockdown GEPs reflect transcriptomic changes by genetic perturbation, which may be correlated to DEPs by drugs targeting the same gene(s).

Second, PPI features were extracted by node2vec method [24] using the PPI network of STRING v11 with 13,592 genes and 297,050 interactions at confidence cutoff ≥ 0.7 [34]. In Node2vec, node features are extracted by applying random walks in a network. Accordingly, this method is designed to preserve local neighborhoods, i.e., mapping neighboring nodes of a network embedded close together in the resulting feature space. 

Third, pathway membership is used to extract target features. Pathway is defined as the collection of functionally related genes, and is a standard tool for functional interpretation including drug MoA [35]. Since pathway member genes are functional related, but not necessarily connected in a PPI network, PM features represent a more global context than PPI-based ones. For feature extraction by PM, we chose Global Vectors for Word Representation (GloVe) by Pennington et al. [25], where GloVe tend to embed targets more closely if they share common pathways more frequently. GloVe is based on word embedding methods, which were also applied to extract gene features using Gene Ontology or coexpression [33,36].

### 3.2. Overall Architecture of DNNs

Our DNN models consist of several blocks of feedforward deep neural networks (FF-DNNs), and both drug and target features are needed as input vectors. Focusing on comparative analysis of target features, we did not try more complex types of DNNs such as RNN (recursive neural network) or CNN (convolutional neural network).

**Figure 1:**
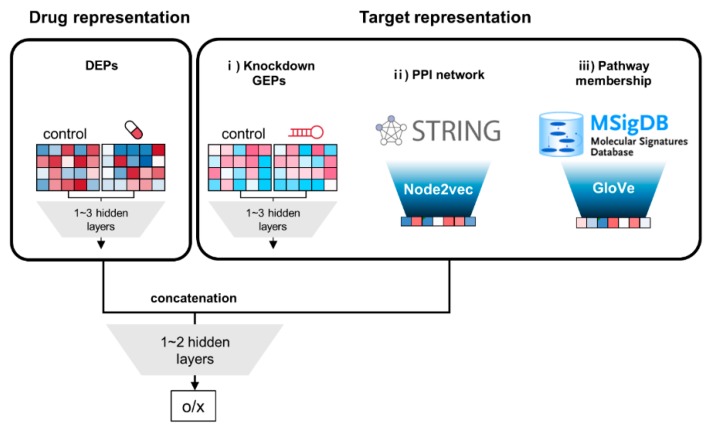
Schematic diagram of deep neural network (DNN) models for comparison of three different target features. First, the model takes two types of input features, i.e., drug and target features, as separate blocks. Drug features are a concatenated vector with 978 + 978 = 1956 elements from both control and treated drug-induced expression profiles (DEPs) from the Library of Integrated Network-based Cellular Signatures (LINCS) L1000 dataset. Target features are obtained from three sources: (i) *knockdown gene expression profiles (GEPs)*; (ii) protein–protein interaction (*PPI) network*; and (iii) *pathway membership*. Target features of GEPs are set to the same structure of 1956-element vectors as drug features. Both features for DEPs and GEPs are connected to 1~3 hidden layers. The target features for the PPI network and pathway membership were extracted by node2vec and Glove method, respectively without hidden layer. The output values of drug and target blocks are concatenated, and subsequently feed to additional 1~2 hidden layers, leading to the final output.

The overall architecture is shown in Figure 1. Their characteristics include: (i) two separate blocks of input features for drug and target, respectively; (ii) the output values of input blocks are concatenated, and additionally connected to 1~2 hidden layers. The input block for drug consists of 1~3 fully connected (FC) hidden layers, which is the same for the target block of GEP. Regarding target features for PPI network and pathway membership, vector representation of genes are already achieved by node2vec and GloVe, respectively. Therefore, their target features are directly employed instead of adding hidden layers. 

### 3.3. Comparison of Target Features 

Then, we compared the prediction performances by the three target features. For objective comparison, the cross-validation & test cycles were performed only for common target space; 965 targets were common to the three type of target features (Figure S1). Therefore, we first tried comparative evaluation of target features limited to the common 965 targets. 

For cross-validation, we collected the DTI dataset from six different DTI databases of ChEMBL Target, Therapeutic Targets Database, MATADOR, KEGG Drug, IUPHAR, PharmGKB, and KiDB [37,38,39,40,41,42,43]. We also collected independent DTI datasets for testing from Binding MOAD and DrugBank [44,45]. DTI information were made mutually exclusive between cross-validation, and test step by allowing any DTI allocated only once. We used known DTIs as positives, and an equal number of unlabeled random pairs as negatives, assuming that positive DTIs are sparse. The list of DTI sources and the full DTI dataset are listed in Tables S2 and S6, respectively.

**Figure 2:**
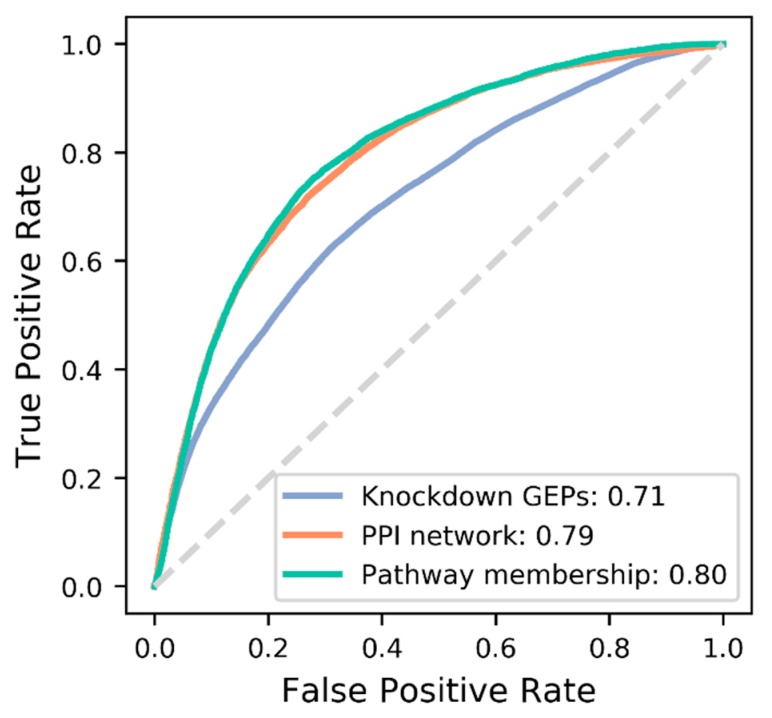
Comparison of prediction performance (AUROC) among the three target features for common DTIs.

We constructed a series of DNNs under different hyperparameter combinations, i.e., number of hidden neurons, activation function, type of target feature, and learning rate (Table S1). Learning parameters were optimized for each target features via 10-fold cross-validation across all 24 parameter combinations (Table S1). The best parameter combination was variable depending on the type of target features (Figure S2). Accordingly, optimized DNNs for each feature were constructed as shown in Figure S3 for final comparison of target features. The DNNs were re-trained using the full training set, and evaluated against the test set (Figure 2). PM and PPI show similar performance of AUROC (Area Under a Receiver Operating Characteristic (ROC) curve) = 0.80 and 0.79, respectively, which are substantially better than GEP (AUROC = 0.71). In order to check the degree of independence among the target features, we calculated the correlations between their distance matrices. PM and PPI seem to be moderately correlated, but uncorrelated with GEP (Figure S4). 

### 3.4. Comparison with DNN and Other Machine Learning Methods

**Figure 3:**
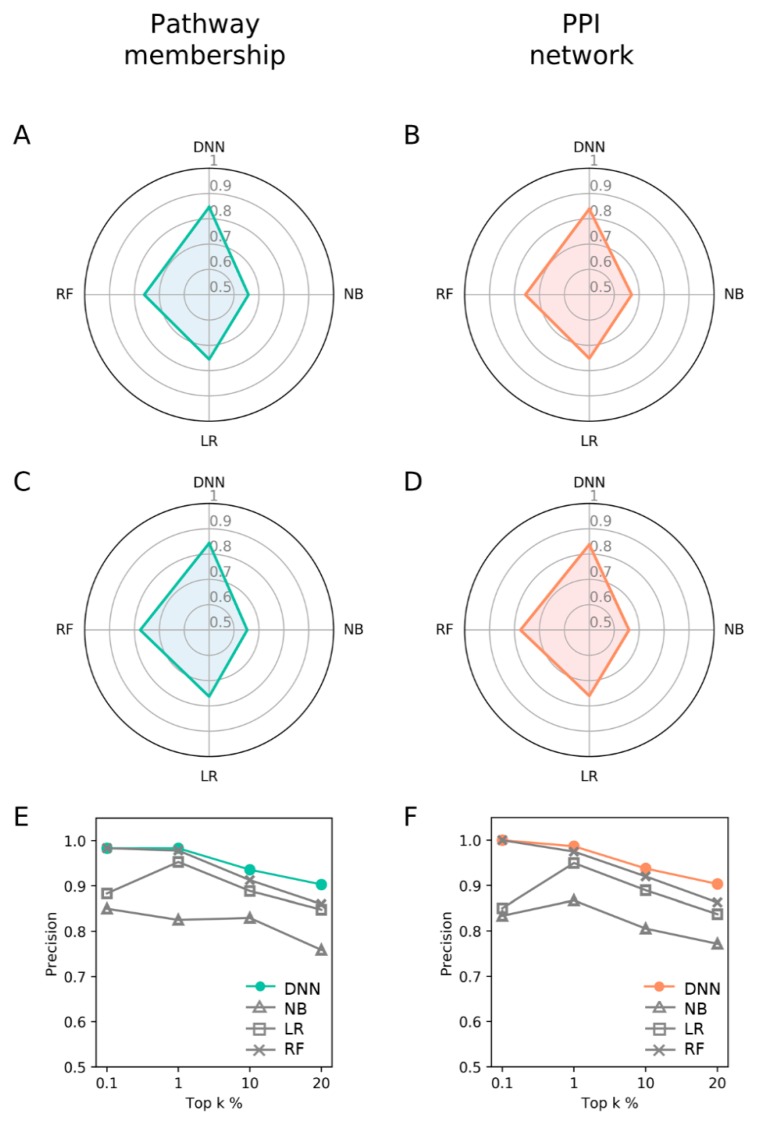
Performance comparison of the DNN models with other machine learning methods. Overall performance comparison by (**A**,**B**) AUROC; and (**C**,**D**) AUPR; (**E**,**F**) The precision for top k% ranked DTIs.

Since PM and PPI showed similarly better performance than GEP, we next compared these two features with other machine learning methods. The comparison was performed in the common target space of 1955 genes between PM and PPI (Figure S1). Accordingly, DNN models were reconstructed and evaluated according to the new target space (Table S3) under a 10-fold cross validation and test scheme. The DNN models were compared with naïve Bayes (NB), logistic regression (LR), and random forest (RF). The machine learning classifiers were trained and evaluated under the same conditions as DNNs. Again, PM and PPI showed similar performance with PM only slightly better. DNNs showed consistently better AUROC (Figure 3A,B) and AUPR (Area under a Precision-Recall curve, Figure 3C,D) than NB, LR, and RF classifiers (Table S4). Such trend is the same with precision at top k% of 0.1~20% (Figure 3E,F). RF models showed comparable performances at top 0.1%~1%. Overall, the performances of DNNs improved compared to those in the previous section (Figure 2 and Figure 3), probably due to expanded training dataset. 

### 3.5. Construction of PM-Based DNN Model Using the Full Dataset 

**Figure 4:**
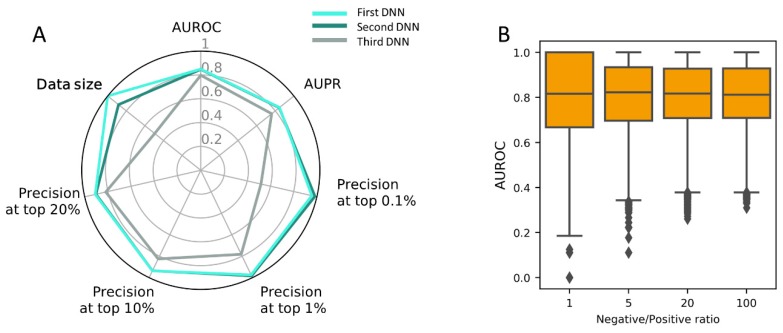
(**A**) Overall performance comparison of the final model by AUROC and the precision for top k% ranked DTIs; (**B**) The influence of negative/positive ratio on the prediction performance of the final DNN model with 576 DTIs as benchmark involving 64 novel compounds from the Comparative Toxicogenomics Database (CTD).

In our experiment, PM was shown best among the three target features tested. Since this model was constructed using a limited dataset, a final model was built using the full dataset (Table S5) under the optimal architecture (Figure S3C). The three PM-based DNNs are of the same architecture and hyperparameters, and different only in terms of the input dataset. It tends to show a better performance with increasing input dataset, although the two DNNs of the largest inputs show little difference (Figure 4A).

In order to evaluate the performance on novel drugs that were not trained during the model-building stage, we collected an additional DTI dataset from the Comparative Toxicogenomics Database (CTD) [46]. We took 576 DTIs for 64 compounds not included in training dataset. In the previous sections, positive and negative dataset was made balanced (e.g., 1:1 ratio) to avoid training bias, which was also frequent in many other related studies for DTI prediction. In reality, positive-negative DTIs are highly skewed toward negatives because positive DTIs are sparse. We also evaluated our DNN model at different negative/positive ratios (1:1 to 100:1), which is expected to better simulate real world situations of sparse DTIs. For each of the 64 compounds, the evaluation was repeated 100 times by sampling negatives 1~100 times the number of positive DTIs, and the median AUROCs were calculated. The distribution of median AUROCs show a robust distribution of 0.82 at varying negative/positive ratios (Figure 4B). 

## 4. Discussion

Recently, the deep neural network (DNN) has been gaining increasing interest as a means of solving complex problems such as protein folding, image-based diagnosis, genomic data interpretation as well as DTI prediction [47,48,49]. DTI prediction is critical for efficient hit discovery, target deconvolution after phenotypic screening, and identifying novel indication in drug repositioning. Drug-induced transcriptome provides rich information on drug MoA, and is considered as an alternative approach for predicting DTIs to modeling-based methods such as docking. For DTI prediction by DNN, we took large-scale DEPs from LINCS L1000 as drug features. 

Unlike other related works based on protein sequences [50,51,52], we used target features reflecting three distinct aspects of protein function, and compared their relative performances. In spite of its preliminary nature, our result suggests that our DNN models consistently outperform several canonical machine learning methods with PM (and PPI) showing the best results. Although network architectures and hyper parameters may not be explored thoroughly, our results may provide useful information for further improvement. Unexpectedly, GEPs showed the worst performance even though DEPs and GEPs were of the same nature, and generated by the same platform. The L1000 dataset has several limitations in that it actually measured only 1000 landmark genes, while those for ~12,000 genes were inferred, and unavailable for the rest ~8000 genes (https://www.biorxiv.org/content/10.1101/332825v2) for both DEPs and GEPs. The performances could be influenced by network type or gene sets for target features from the PPI network or pathway membership, respectively. Most of all, our DNN model may not have been trained sufficiently due to a limited number of DTI training data. This suggests that there is plenty of room for improvement with increasing dataset and integration of different features.

## Supplementary Materials

The following are available online at https://www.mdpi.com/1999-4923/11/8/377/s1, Figure S1: Target coverage by the three target features of PPI network, knockdown GEPs, and pathway membership, where the number of associated unique targets is indicated; Figure S2: Performance distribution depending on each hyper parameter of (**A**) Activation function before concatenation; (**B**) Number of hidden neurons for drug features (DEPs); (**C**) Number of hidden neurons after concatenation; and (**D**) Learning rate. The best parameter combination was selected showing the minimum median of loss in 10-fold cross-validation for each parameter and feature type; Figure S3: Optimized architecture of DNN models for (**a**) knockdown GEPs; (**b**) PPI network; and (**c**) pathway membership; Figure S4: The degree of Pearson (Spearman) correlations among the distance matrices of target features; Table S1. Hyper parameters for building DNN models; Table S2: Data sources for preparing training, validation, and test dataset; Table S3: A common dataset of PM and PPI to compare DNN and other machine learning; Table S4: Performance comparison between DNN and other machine learning in AUROC (AUPR); Table S5: Datasets for training and testing the final DNN model. Table S6: The list of DTI dataset for training and testing.

## References

[1] Senior A., Jumper J., Hassabis D. AlphaFold: Using AI for Scientific Discovery. https://deepmind.com/blog/alphafold/.

[2] Hopkins A.L. (2008). Network pharmacology: The next paradigm in drug discovery. Nat. Chem. Biol..

[3] Rifaioglu A.S., Atas H., Martin M.J., Cetin-Atalay R., Atalay V., Do T. (2018). Recent applications of deep learning and machine intelligence on in silico drug discovery: Methods, tools and databases. Brief. Bioinform..

[4] Chen X., Yan C.C., Zhang X., Zhang X., Dai F., Yin J., Zhang Y. (2016). Drug–target interaction prediction: Databases, web servers and computational models. Brief. Bioinform..

[5] Ding H., Takigawa I., Mamitsuka H., Zhu S. (2014). Similarity-based machine learning methods for predicting drug–target interactions: A brief review. Brief. Bioinform..

[6] Ripphausen P., Nisius B., Bajorath J. (2011). State-of-the-art in ligand-based virtual screening. Drug Discov. Today.

[7] Lionta E., Spyrou G., Vassilatis D.K., Cournia Z. (2014). Structure-based virtual screening for drug discovery: Principles, applications and recent advances. Curr. Top. Med. Chem..

[8] Drwal M.N., Griffith R. (2013). Combination of ligand- and structure-based methods in virtual screening. Drug Discov. Today Technol..

[9] Ezzat A., Wu M., Li X.-L., Kwoh C.-K. (2018). Computational prediction of drug–target interactions using chemogenomic approaches: An empirical survey. Brief. Bioinform..

[10] Chen R., Liu X., Jin S., Lin J., Liu J. (2018). Machine Learning for Drug-Target Interaction Prediction. Molecules.

[11] Lamb J., Crawford E.D., Peck D., Modell J.W., Blat I.C., Wrobel M.J., Lerner J., Brunet J.-P., Subramanian A., Ross K.N. (2006). The Connectivity Map: Using Gene-Expression Signatures to Connect Small Molecules, Genes, and Disease. Science.

[12] Musa A., Ghoraie L.S., Zhang S.-D., Glazko G., Yli-Harja O., Dehmer M., Haibe-Kains B., Emmert-Streib F. (2018). A review of connectivity map and computational approaches in pharmacogenomics. Brief. Bioinform..

[13] Iorio F., Bosotti R., Scacheri E., Belcastro V., Mithbaokar P., Ferriero R., Murino L., Tagliaferri R., Brunetti-Pierri N., Isacchi A. (2010). Discovery of drug mode of action and drug repositioning from transcriptional responses. Proc. Natl. Acad. Sci. USA.

[14] Wang K., Sun J., Zhou S., Wan C., Qin S., Li C., He L., Yang L. (2013). Prediction of drug-target interactions for drug repositioning only based on genomic expression similarity. PLoS Comput. Biol..

[15] Hizukuri Y., Sawada R., Yamanishi Y. (2015). Predicting target proteins for drug candidate compounds based on drug-induced gene expression data in a chemical structure-independent manner. BMC Med. Genom..

[16] Isik Z., Baldow C., Cannistraci C.V., Schroeder M. (2015). Drug target prioritization by perturbed gene expression and network information. Sci. Rep..

[17] Subramanian A., Narayan R., Corsello S.M., Peck D.D., Natoli T.E., Lu X., Gould J., Davis J.F., Tubelli A.A., Asiedu J.K. (2017). A Next Generation Connectivity Map: L1000 Platform and the First 1,000,000 Profiles. Cell.

[18] Van Noort V., Scholch S., Iskar M., Zeller G., Ostertag K., Schweitzer C., Werner K., Weitz J., Koch M., Bork P. (2014). Novel Drug Candidates for the Treatment of Metastatic Colorectal Cancer through Global Inverse Gene-Expression Profiling. Cancer Res..

[19] Li H.-D., Guan Y., Burmeister M., Zhang H., Wall D.P., Duda M. (2018). Brain-specific functional relationship networks inform autism spectrum disorder gene prediction. Transl. Psychiatry.

[20] Brum A.M., van de Peppel J., van der Leije C.S., Schreuders-Koedam M., Eijken M., van der Eerden B.C.J., van Leeuwen J.P.T.M. (2015). Connectivity Map-based discovery of parbendazole reveals targetable human osteogenic pathway. Proc. Natl. Acad. Sci. USA.

[21] Lee H., Kang S., Kim W. (2016). Drug Repositioning for Cancer Therapy Based on Large-Scale Drug-Induced Transcriptional Signatures. PLoS ONE.

[22] Iwata M., Sawada R., Iwata H., Kotera M., Yamanishi Y. (2017). Elucidating the modes of action for bioactive compounds in a cell-specific manner by large-scale chemically-induced transcriptomics. Sci. Rep..

[23] Xie L., He S., Song X., Bo X., Zhang Z. (2018). Deep learning-based transcriptome data classification for drug-target interaction prediction. BMC Genom..

[24] Grover A., Leskovec J. (2016). Node2vec.

[25] Pennington J., Socher R., Manning C. (2014). Glove: Global Vectors for Word Representation. Proceedings of the 2014 Conference on Empirical Methods in Natural Language Processing (EMNLP).

[26] Liberzon A., Birger C., Thorvaldsdóttir H., Ghandi M., Mesirov J.P., Tamayo P. (2015). The Molecular Signatures Database Hallmark Gene Set Collection. Cell Syst..

[27] Subramanian A., Tamayo P., Mootha V.K., Mukherjee S., Ebert B.L., Gillette M.A., Paulovich A., Pomeroy S.L., Golub T.R., Lander E.S. (2005). Gene set enrichment analysis: A knowledge-based approach for interpreting genome-wide expression profiles. Proc. Natl. Acad. Sci. USA.

[28] Abadi M., Agarwal A., Barham P., Brevdo E., Chen Z., Citro C., Corrado G.S., Davis A., Dean J., Devin M. (2016). TensorFlow: Large-Scale Machine Learning on Heterogeneous Distributed Systems. arXiv.

[29] Chollet F. (2015). Keras. https://github.com/fchollet/keras.

[30] Kingma D.P., Ba J. (2014). Adam: A Method for Stochastic Optimization. arXiv.

[31] Srivastava N., Hinton G., Krizhevsky A., Salakhutdinov R. (2014). Dropout: A Simple Way to Prevent Neural Networks from Overfitting. J. Mach. Learn. Res..

[32] Peng J., Guan J., Shang X. (2019). Predicting Parkinson’s Disease Genes Based on Node2vec and Autoencoder. Front. Genet..

[33] Smaili F.Z., Gao X., Hoehndorf R. (2018). Onto2Vec: Joint vector-based representation of biological entities and their ontology-based annotations. Bioinformatics.

[34] Szklarczyk D., Morris J.H., Cook H., Kuhn M., Wyder S., Simonovic M., Santos A., Doncheva N.T., Roth A., Bork P. (2017). The STRING database in 2017: Quality-controlled protein–protein association networks, made broadly accessible. Nucleic Acids Res..

[35] Chen L., Chu C., Lu J., Kong X., Huang T., Cai Y.-D. (2015). Gene Ontology and KEGG Pathway Enrichment Analysis of a Drug Target-Based Classification System. PLoS ONE.

[36] Du J., Jia P., Dai Y., Tao C., Zhao Z., Zhi D. (2019). Gene2vec: Distributed representation of genes based on co-expression. BMC Genom..

[37] Chen X., Ji Z.L., Chen Y.Z. (2002). TTD: Therapeutic Target Database. Nucleic Acids Res..

[38] Davies M., Nowotka M., Papadatos G., Dedman N., Gaulton A., Atkinson F., Bellis L., Overington J.P. (2015). ChEMBL web services: Streamlining access to drug discovery data and utilities. Nucleic Acids Res..

[39] Gunther S., Kuhn M., Dunkel M., Campillos M., Senger C., Petsalaki E., Ahmed J., Urdiales E.G., Gewiess A., Jensen L.J. (2007). SuperTarget and Matador: Resources for exploring drug-target relationships. Nucleic Acids Res..

[40] Kanehisa M., Furumichi M., Tanabe M., Sato Y., Morishima K. (2017). KEGG: New perspectives on genomes, pathways, diseases and drugs. Nucleic Acids Res..

[41] Sharman J.L., Benson H.E., Pawson A.J., Lukito V., Mpamhanga C.P., Bombail V., Davenport A.P., Peters J.A., Spedding M., Harmar A.J. (2013). IUPHAR-DB: Updated database content and new features. Nucleic Acids Res..

[42] Thorn C.F., Klein T.E., Altman R.B. (2013). PharmGKB: The Pharmacogenomics Knowledge Base. Methods Mol. Biol..

[43] Roth B.L., Lopez E., Patel S., Kroeze W.K. (2000). The Multiplicity of Serotonin Receptors: Uselessly Diverse Mol ecules or an Embarrassment of Riches?. Neuroscientist.

[44] Ahmed A., Smith R.D., Clark J.J., Dunbar J.B., Carlson H.A. (2015). Recent improvements to Binding MOAD: A resource for protein–ligand binding affinities and structures. Nucleic Acids Res..

[45] Law V., Knox C., Djoumbou Y., Jewison T., Guo A.C., Liu Y., Maciejewski A., Arndt D., Wilson M., Neveu V. (2014). DrugBank 4.0: Shedding new light on drug metabolism. Nucleic Acids Res..

[46] Davis A.P., Murphy C.G., Johnson R., Lay J.M., Lennon-Hopkins K., Saraceni-Richards C., Sciaky D., King B.L., Rosenstein M.C., Wiegers T.C. (2013). The Comparative Toxicogenomics Database: Update 2013. Nucleic Acids Res..

[47] Alipanahi B., Delong A., Weirauch M.T., Frey B.J. (2015). Predicting the sequence specificities of DNA- and RNA-binding proteins by deep learning. Nat. Biotechnol..

[48] Zhou J., Troyanskaya O.G. (2015). Predicting effects of noncoding variants with deep learning–based sequence model. Nat. Methods.

[49] Webb S. (2018). Deep learning for biology. Nature.

[50] Öztürk H., Özgür A., Ozkirimli E. (2018). DeepDTA: Deep drug-target binding affinity prediction. Bioinformatics.

[51] Feng Q., Dueva E., Cherkasov A., Ester M. (2018). PADME: A Deep Learning-based Framework for Drug-Target Interaction Prediction. arXiv.

[52] Wen M., Zhang Z., Niu S., Sha H., Yang R., Yun Y., Lu H. (2017). Deep-Learning-Based Drug−Target Interaction Prediction. J. Proteome Res..

