# Supplementary material for: Comparison of Target Features for Predicting Drug-Target Interactions by Deep Neural Network Based on Large-Scale Drug-Induced Transcriptome Data
Source: Pharmaceutics. 2019 Aug 2;11(8):377. doi: 10.3390/pharmaceutics11080377 (PMC6723794; doi:10.3390/pharmaceutics11080377)
Supplement: Supplementary file 1 [file pharmaceutics-11-00377-s001.zip › pharmaceutics-536783-supplementary-for conversion/Supplementary_material.docx]

Supplementary Materials: Comparison of Target Features for Predicting Drug-Target Interactions by Deep Neural Network Based on Large-Scale Drug-Induced Transcriptome Data

Hanbi Lee and Wankyu Kim


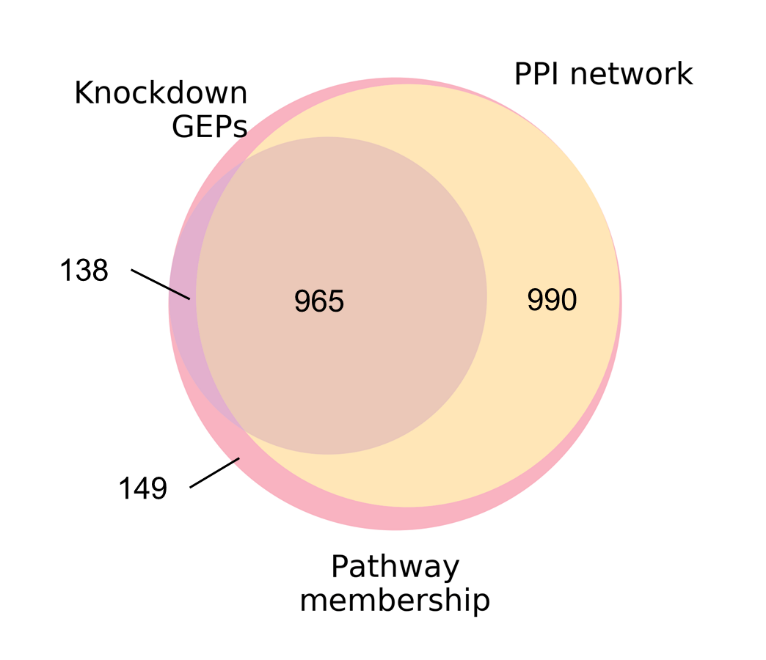


**Figure S1.** Target coverage by the three target features of PPI network, knockdown GEPs, and pathway membership, where the number of associated unique targets is indicated.


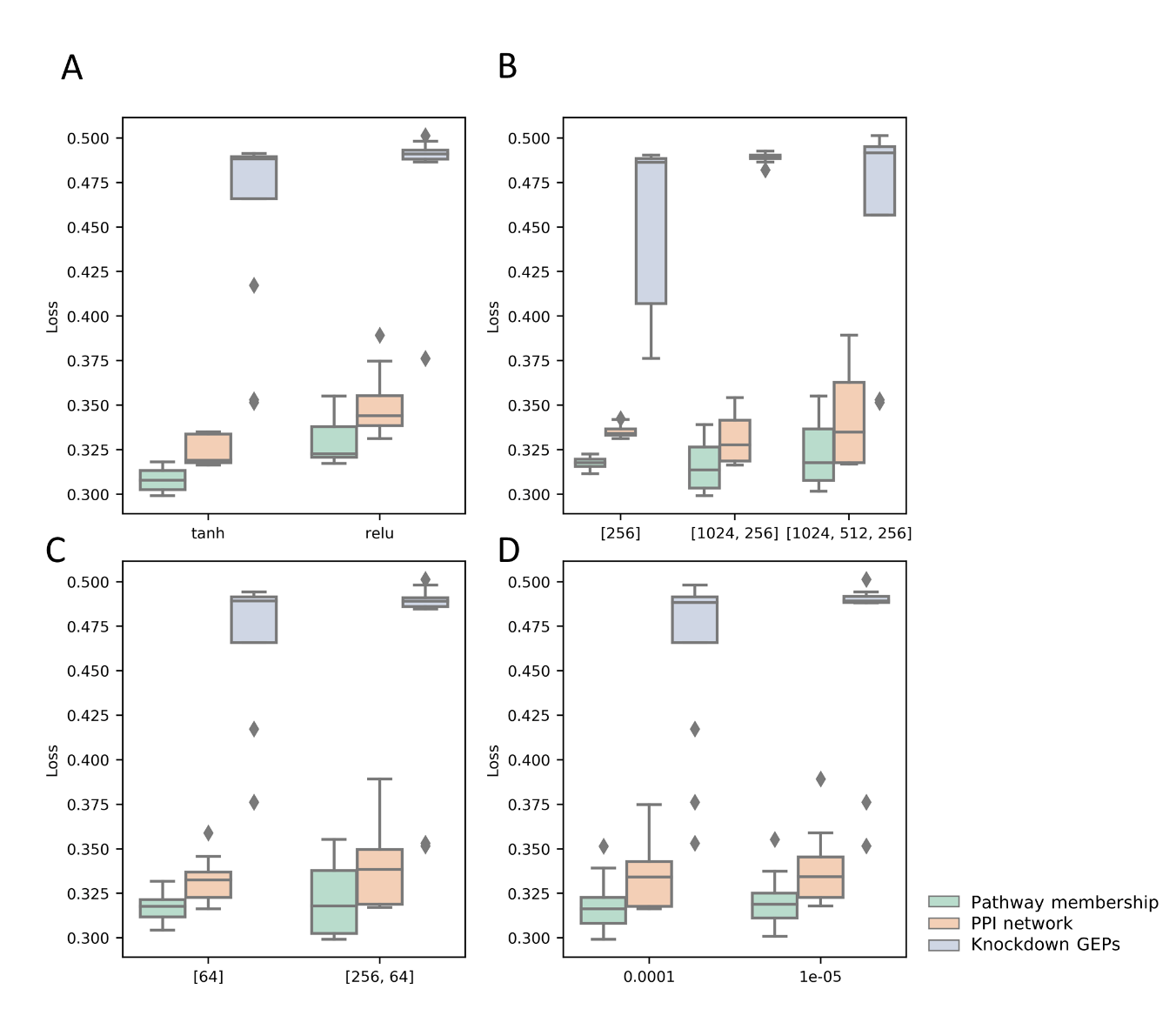


**Figure S2.** Performance distribution depending on each hyper parameter of A) Activation function before concatenation, B) Number of hidden neurons for drug features (DEPs), C) Number of hidden neurons after concatenation, and D) Learning rate. The best parameter combination was selected showing the minimum median of loss in 10-fold cross-validation for each parameter and feature type.


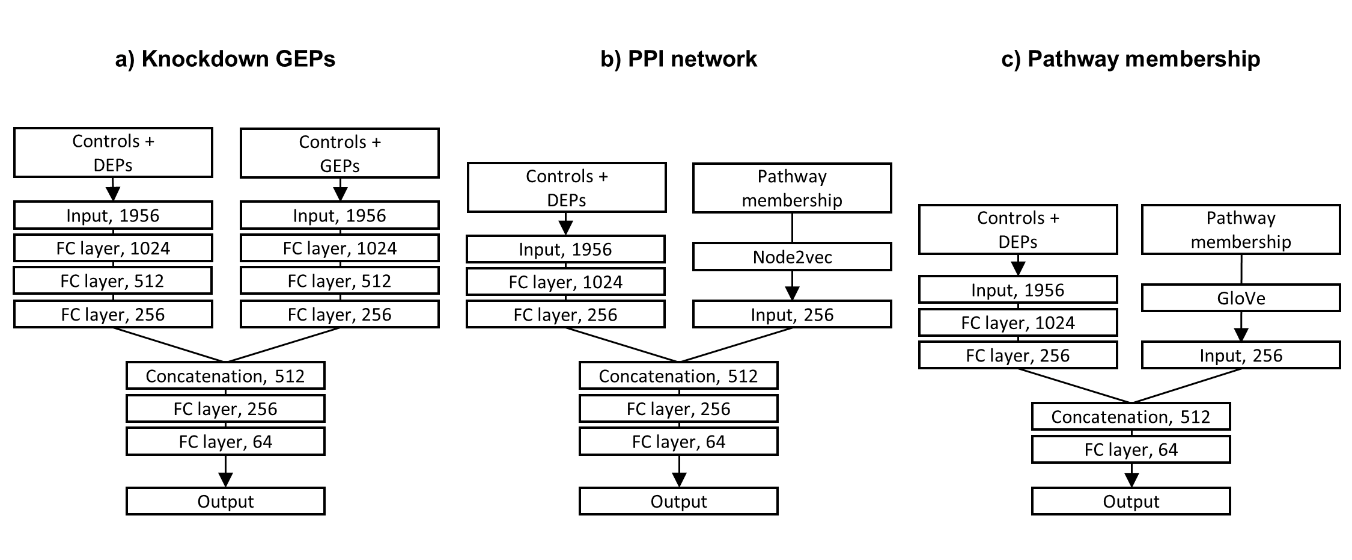


**Figure S3.** Optimized architecture of DNN models for (**a**) knockdown GEPs, (**b**) PPI network and (**c**) pathway membership.


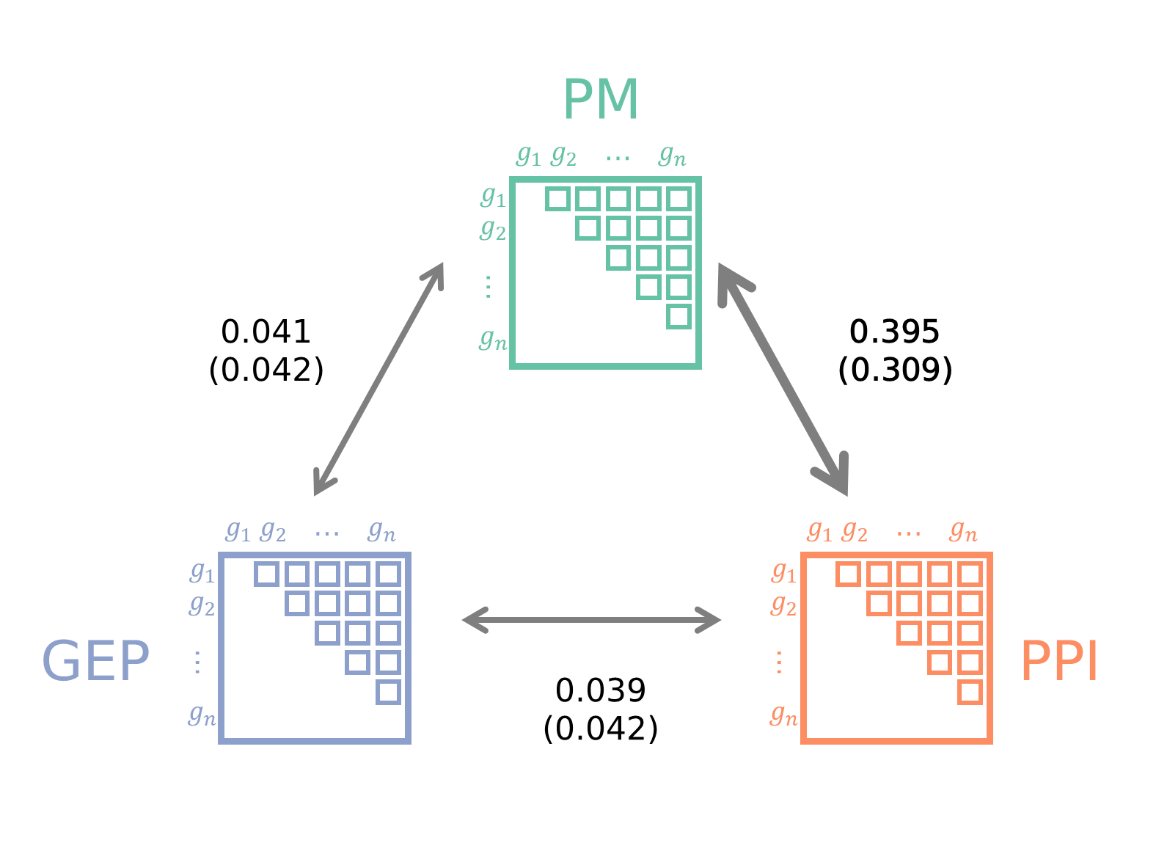


**Figure S4.** The degree of Pearson (Spearman) correlations among the distance matrices of target features. The distance matrix was calculated as pairwise cosine distances among the target features of common genes (*n* = 965) by PM, PPI, and GEP. In the case that multiple features exist for a single gene as in GEP, we took the median value of all-pairwise distances for a gene pair.

**Table S1.** Hyper parameters for building DNN models.

| **Title 1** | **Title 2** |
| --- | --- |
| Hidden neurons for drug features (DEPs) and knockdown GEPs | [1024, 512, 256],  [1024, 256],  [2565] |
| Activation function before concatenation | Tanh, ReLu |
| Activation function after concatenation | ReLu |
| Hidden neurons after concatenation | [256, 64],  [64] |
| Learning rate | 0.0001,  0.00001 |
| Dropout | 0.5 |

**Table S2.** Data sources for preparing training, validation, and test dataset.

|  | **Training and Validation** | **Test** |
| --- | --- | --- |
| DTI data sources | Chembl Target, KiDB, MATADOR, IUPHAR, KEGG Drug, PharmGKB, Therapeutic Targets Database | Binding MOAD, DrugBank |
| Unique Drugs | 1459 | 392 |
| Drug-induced Expression Profiles (DEPs) | 24,158 | 7360 |
| Unique Targets | 881 | 282 |
| Target Features by Gene Knockdown (GEPs) | 7694 | 2674 |
| Unique pairs of DTIs | 8575 | 694 |

**Table S3.** A common dataset of PM and PPI to compare DNN and other machine learning.

|  | **Training** | **Test** |
| --- | --- | --- |
| Databases | Chembl Target, KiDB, MATADOR, IUPHAR, KEGG Drug, PharmGKB, Therapeutic Targets Database | Binding MOAD, DrugBank |
| Unique Drugs | 1811 | 609 |
| Drug-induced Expression Profiles (DEPs) | 28,037 | 9701 |
| Unique Targets | 1788 | 481 |
| Unique pairs of DTIs | 17,058 | 1618 |

**Table S4.** Performance comparison between DNN and other machine learning in AUROC (AUPR).

|  | **DNN** | **LR** | **NB** | **RF** |
| --- | --- | --- | --- | --- |
| PPI network | 0.838  (0.838) | 0.752  (0.760) | 0.670  (0.660) | 0.757  (0.776) |
| Pathway membership | 0.849  (0.846) | 0.755  (0.763) | 0.658  (0.653) | 0.760  (0.777) |

**Table S5.** Datasets for training and testing the final DNN model.

|  | **Training** | **Test** |
| --- | --- | --- |
| Databases | Chembl Target, KiDB, MATADOR, IUPHAR, KEGG Drug, PharmGKB, Therapeutic Targets Database | Binding MOAD, DrugBank |
| Unique Drugs | 1838 | 656 |
| Drug-induced Expression Profiles (DEPs) | 28,380 | 10,314 |
| Unique Targets | 2060 | 539 |
| Unique pairs of DTIs | 19,206 | 1863 |

**Table 6.** The list of DTI dataset for training and testing.

The table content could be found at attached the Excel file.
